# QTL Mapping and Candidate Gene Screening for Enhancing Oil Content in Silage Maize

**DOI:** 10.3390/plants14081181

**Published:** 2025-04-10

**Authors:** Jianzhong Wu, Qi Wang, Weibo Han, Qian Zhao, Dequan Sun, Zhongbao Shen

**Affiliations:** 1 Institute of Forage and Grassland Sciences, Heilongjiang Academy of Agricultural Sciences, Harbin 150086, China; wujianzhong176@163.com (J.W.); alclever@163.com (W.H.); 2 College of Life Science and Technology, Harbin Normal University, Harbin 150025, China; m18846603788@163.com; 3 Cultivation and Farming Research Institute, Heilongjiang Academy of Agricultural Sciences, Harbin 150086, China; zhaoqian0401@sina.com

**Keywords:** silage maize, oil content, QTL, transcriptome, gene mining

## Abstract

Assessing the nutritional quality of silage maize (*Zea mays* L.) hinges largely on its oil content, a complex quantitative trait influenced by multiple genes. Mining candidate genes within oil content-related quantitative trait loci (QTLs) can provide genetic resources and a theoretical foundation for cultivating high-oil silage maize varieties. This study employed 274 doubled haploid (DH) lines derived from the parental lines BY4944 and DNF34-2 to perform main gene plus polygene mixed genetic analysis and complex interval mapping (CIM), with the goal of pinpointing oil content-related QTLs and genes distributed across the *Z. mays* L. genome. Leveraging 5400 single nucleotide polymorphism (SNPs), a high-resolution silage maize genetic linkage map covering 3864.51 cM was constructed with an average interval between markers of 0.74 cM. Analysis of the map revealed 13 oil content-related QTLs. The most significant large-effect QTL (qOIL-1-1), located on chromosome 1 within the region spanning 240.93 Mb to 256.57 Mb, exhibited a logarithm of odds (LOD) score of 3.34 and explained 5.06% of oil content-related phenotypic variation. Within these QTLs, 617 genes were annotated. Through transcriptome analysis combined with quantitative real-time polymerase chain reaction (RT-qPCR), five candidate genes potentially associated with oil content were predicted and subsequently validated within these genetic loci. This research underscores the potential of identifying candidate genes to enhance breeding efforts aimed at augmenting oil content, thereby advancing animal husbandry practices.

## 1. Introduction

Corn silage (*Zea mays* L.) is renowned for its high nutritional value, palatability, and digestibility, serving as a staple component of livestock feed. Its global cultivation area ranks consistently among the top three forages worldwide [1]. Its proven ability to enhance both livestock production efficiency and quality underscores its pivotal role in driving economic prosperity within the industry [2]. With the rapid expansion of animal husbandry and the dairy sector, there is a pressing need to further enhance the nutritional attributes of corn silage to meet the escalating demand for high-quality feed.

Oil content plays vital roles in various biological processes associated with plant growth and development. During the ensiling process of high-oil maize, specific lipid constituents undergo enzymatic conversion into nutritionally advanced fatty acids. These bioactive compounds enhance livestock and poultry health indices, including growth performance parameters (body weight gain, feed efficiency) and developmental biomarkers, while concurrently improving the nutritional profile of livestock products [3]. Compared to conventional corn, high-oil corn exhibits superior energy density and nutritional composition, which enables reduced daily feed consumption when utilized as animal feedstock [4]. Furthermore, high-oil corn varieties demonstrate desirable agronomic characteristics, including enhanced plant vigor, elevated biomass production, and robust stress tolerance [5].

Quantitative trait loci (QTL) mapping serves as the cornerstone of molecular breeding and plays a pivotal role in facilitating and accelerating the development of improved crops. The identification of chromosomal regions associated with oil content-related traits through QTL mapping contributes to the development of maize germplasm with diverse genetic backgrounds. For example, Yang et al. [6] identified 42 QTLs located in 21 genomic regions governing fatty acid composition and grain oil content that explained between 0.7 and 48.3% of the phenotypic variation in these traits, respectively. In addition, Lin et al. [7] identified 70 QTL clusters related to corn kernel oil content spanning 34.5% of the maize genome, with 59 (84%) QTL clusters co-isolated with at least one gene within the set of 147 oil-related genes. Furthermore, Fang et al. [8] detected a total of 62 QTLs related to oil content traits, with contribution rates ranging from 12.5% to 52.5%, while Yang [9] et al. detected a total of 11 oil-related QTLs, including a single QTL explaining 4.6% to 11.1% of the phenotypic variation in this trait. In a separate study, Yu et al. [10] detected a total of nine oil content-related conditional QTLs with contribution rates ranging from 2.4 to 20.6%.

Innovations in genomic technologies have propelled quantitative trait genetic dissection into a new era, where high-density molecular markers combined with genome-wide association-based QTL mapping approaches have emerged as the central strategy for dissecting the genetic basis of complex agronomic traits [11]. Integrated QTL–transcriptome analysis can reduce the candidate gene screening scope by 75–90%, thereby significantly enhancing functional validation success rates, as evidenced in crops such as cotton [12], maize [13], and rapeseed [14].

Studies have shown that in corn, a low-oil seed crop, only 20.1% of oil-related genes are regulated. Moreover, the corn genome contains fewer oil biosynthesis genes compared to genomes of high-oil seeds [15]. Notably, while many maize genes share homology with regulated genes in other seed oil crops, most maize genes encoding oil-associated transcription factors, triacylglycerol (TAG) biosynthetase, pentose phosphate pathway (PPP), and Calvin cycle proteins are not regulated during seed oil synthesis [16]. These findings underscore the intricate genetic regulation underlying maize oil content and emphasize the critical importance of developing silage-specific high-oil corn hybrids for advancing livestock industry development.

Herein, we established a population of 274 double haploid (DH) lines derived from parental lines (BY4944 and DNF34-2) exhibiting significant oil content divergence. A high-density genetic linkage map was constructed using composite interval mapping (CIM) to identify significant QTLs. Candidate genes associated with oil content were predicted and validated through transcriptome analysis combined with RT-qPCR. This study provides a theoretical foundation for elucidating the genetic basis of oil content in silage maize and guides the targeted selection of elite germplasms for developing varieties with high-oil kernels.

## 2. Results

### 2.1. Analysis of Silage Maize Grain Phenotypic Variation

**Figure 1:**
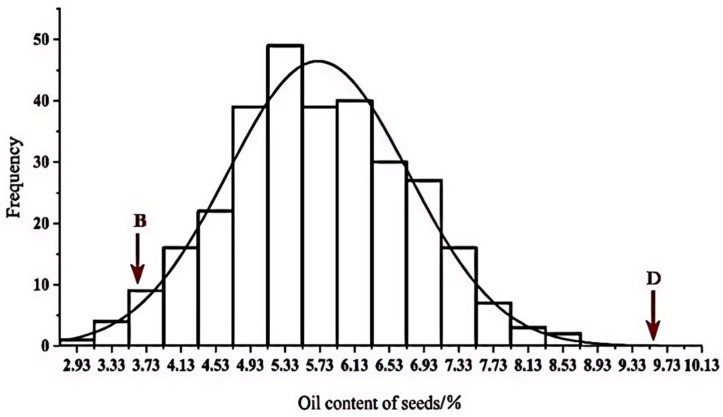
Histogram of frequency distribution of traits in DH line. B stands for maternal BY4944 and D stands for paternal DNF34-2.

The mean oil content of the DH population was 5.72, with a standard deviation of 1.07 and an environmental variance of 0.14. The coefficient of variation was calculated at 18.25%, indicating significant potential for genetic enhancement within this trait. Subsequent Kolmogorov–Smirnov (K-S) testing revealed a Sig value (*p*-value) for oil content exceeding 0.05 (Table 1), indicating conformity with a normal distribution pattern (Figure 1).

Phenotypic values of the parents were found at the two extremes of the population distribution. Notably, the average phenotypic value of the DH population was lower than that of the median parent, indicating a negative transgressive inheritance. However, the oil content traits displayed a tendency to align more closely with those of the male parent.

### 2.2. Sequencing and SNP Identification

The raw reads obtained from the 274 DH lines exhibited substantial variation, ranging from 60.25 Mb to 772.37 Mb, with a mean of 449.63 Mb. The 1× genome coverage spanned 82.02%~91.94% (mean 90.03%), while 4× genome coverage ranged between 81.93~94.22% (mean 88.56%). The parental lines BY4944 and DNF34-2 generated 376.35 Mb and 398.73 Mb raw reads, respectively, demonstrating 1× coverage of 96.95% and 97.21%, and 4× coverage reaching 94.27% and 96.11%, correspondingly. After filtering, the clean reads from the DH population exhibited genome coverage ranging from 54.37 Mb to 706.31 Mb, with an average coverage of 413.82 Mb; the genome coverage values of clean reads from the BY4944 and DNF34-2 parental lines were 348.75 Mb and 371.53 Mb, respectively. The Q20 values for the clean reads of the DH population ranged from 97.21% to 99.13%, with an average value of 98.38%; the Q20 values of BY4944 and DNF34-2 were 99.27% and 99.20%, respectively (Table S1).

**Figure 2:**
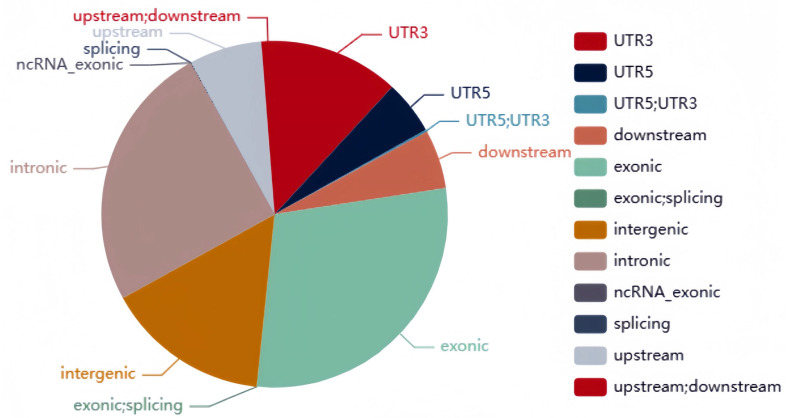
Distribution of core SNPs.

A total of 51,943 polymorphic SNP molecular markers were identified, with 14,510 of them belonging to type aa × bb. After progeny polymorphism filtering, 5400 SNPs distributed across the ten maize chromosomes were identified, with SNP numbers per chromosome ranging from 302 to 1014 (Table 2, Figure S1). The genome coverage of the DH population of SNPs ranged from 82.02% to 91.94% (mean 90.03%), while that of the BY4944 and DNF34-2 parental lines covered 93.12% and 93.24% of the genome, respectively (Table S1). Core SNP sites were mainly found within exonic (29.16%), intronic (24.94%), and intergenic (15.3%) regions (Figure 2).

### 2.3. Construction of Genetic Linkage Map

**Figure 3:**
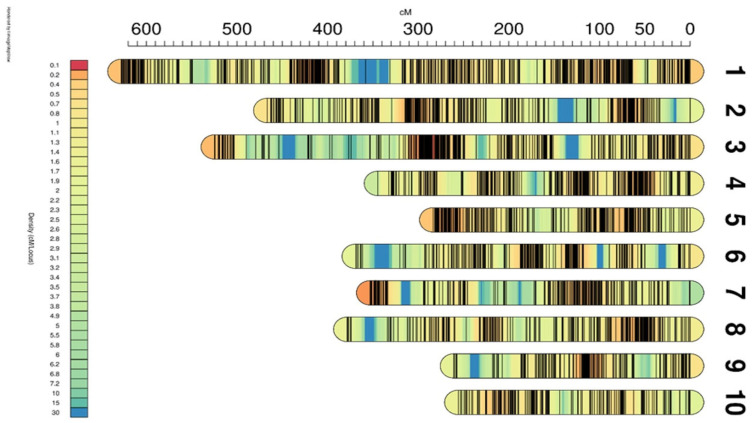
Genetic linkage map of silage maize. The map shows the position of each marker on the genetic map, the heat map represents the density of the marker on the genetic map, and the color per 1 cM represents the value of 30 cM divided by the number of genetic markers in the 15 cM range of the upper and lower reaches of the 1 cM.

We successfully constructed a genetic linkage map comprising 10 linkage groups (LG01-LG10), with a total length of 3864.51 cM (Table 2, Figure 3). The genetic distances between the 10 linkage groups ranged from 255.91 cM to 627.74 cM, with the average distances between markers ranging from 0.49 cM to 0.95 cM. Notably, the maximum distance between markers within LG03 was 42.76 cM and between markers within LG10 was 13.06 cM. Furthermore, we observed consistent collinearity between the 5400 markers and their physical locations in the reference genome (Figure S2), confirming the high accuracy and reliability of our constructed genetic linkage map.

### 2.4. Mapping of Silage Maize QTLs

**Figure 4:**
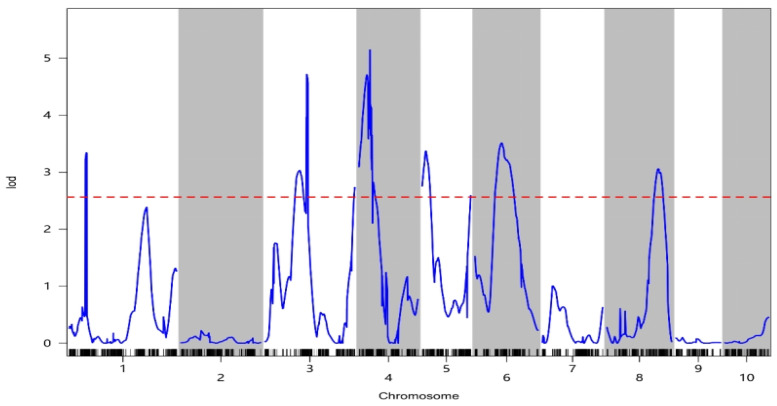
LOD curve of oil content characteristics. The horizontal coordinate is the genetic position of the chromosome (linked group), the vertical coordinate is the LOD value, the blue curve is the LOD value curve, the short black vertical line below the blue line is the location of the marker, and the red dashed line is the threshold line.

In this study, we identified 13 QTLs associated with oil content distributed across chromosomes 1, 3, 4, 5, 6, and 8, respectively (Table 3). The LOD scores ranged from 2.59 to 5.15, indicating the high statistical significance of these QTLs (Figure 4), and the contribution rates indicating explained phenotype variation rates ranged from 0.01% to 5.06%.

Among these QTLs, qOIL-1-1 emerged as noteworthy, with an LOD value of 3.34 and a physical interval spanning 240.93 to 256.57 Mb on chromosome 1. This QTL accounted for the largest effect on oil content, explaining 5.06% of the phenotypic variation in this trait. Therefore, this QTL was hypothesized to be a main effect QTL, with additive effect values ranging from −0.25 to −0.22.

### 2.5. Prediction of Candidate Genes Within QTL Intervals Associated with Silage Maize Oil Content Traits

**Figure 5:**
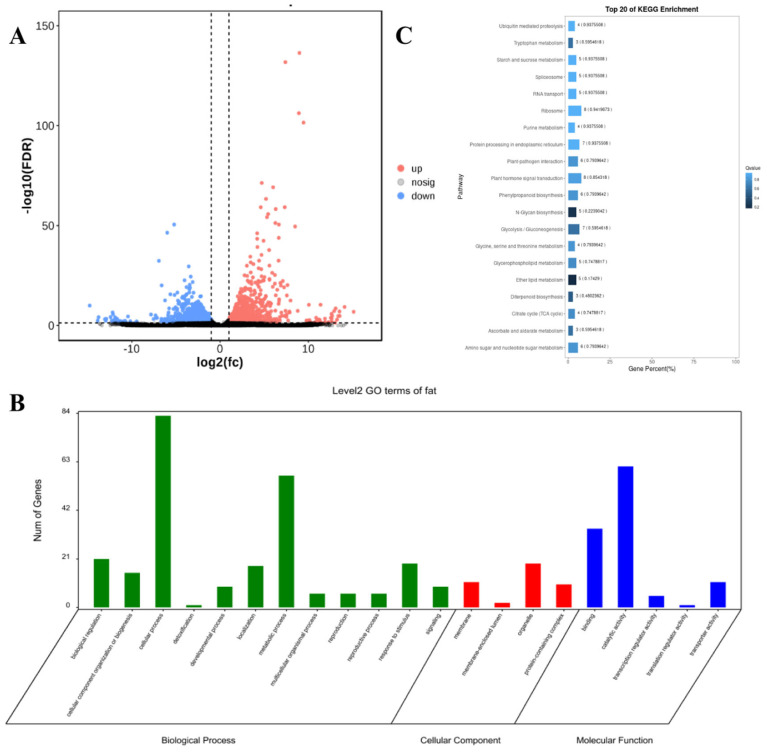
(**A**) volcanic map of differentially expressed genes related to oil content traits. The horizontal axis represents the degree of upregulation or downregulation, the vertical axis represents the number of genes, red represents upregulated genes, and blue represents downregulated genes; (**B**) GO enrichment analysis results of differentially expressed genes related to oil content; (**C**) the top 20 pathways for KEGG enrichment analysis of differentially expressed genes related to oil content.

The correlation coefficients (γ) among biological replicates within both the OH and OL groups exceeded 0.9, indicating strong inter-sample consistency and experimental reproducibility (Figure S3). Comparative transcriptomic analysis identified 981 differentially expressed genes (DEGs), with 549 upregulated and 432 downregulated in OH compared to OL (Figure 5A). Gene Ontology (GO) enrichment revealed 617 DEGs (*p* < 0.05) significantly clustered into 21 subcategories across three major domains: biological process (BP), cellular component (CC), and molecular function (MF). BP dominated the functional annotations, encompassing the highest proportion of terms, including RNA biosynthetic process (GO:0032774), inositol phosphate biosynthetic process (GO:0010919), and regulation of lipase activity (GO:0060191). CC annotations highlighted plasma membrane (GO:0005887), organelle inner membrane (GO:0031226), and cytoplasmic vesicle (GO:0031410), while MF terms featured ligase activity (GO:0016874), α-1,2-mannosyltransferase activity (GO:0000026), and geranylgeranyltransferase activity (GO:0004337) (Figure 5B).

KEGG pathway analysis mapped all differentially expressed genes (DEGs) to 113 metabolic pathways (Figure 5C). The top 20 enriched pathways included ether lipid metabolism (ko00565), diterpenoid biosynthesis (ko00904), and critical lipid-related pathways such as α-linolenic acid metabolism (ko00592) and glycerophospholipid metabolism (ko00564). Redundancy filtering identified 23 unique DEGs functionally linked to these lipid synthesis pathways.

To refine candidate selection, 232 significantly differential genes were screened from QTL-associated linkage groups. Physical alignment of these genes with QTL intervals identified five oil biosynthesis candidates (Table 4): GRMZM2G133398 and GRMZM2G156861 within qOIL-3-2, GRMZM2G125268 (qOIL-4-2), GRMZM2G002959 (qOIL-6-2), and GRMZM2G343588 (qOIL-8-1).

### 2.6. Validation of Oil Content-Associated Differentially Expressed Genes via RT-qPCR

**Figure 6:**
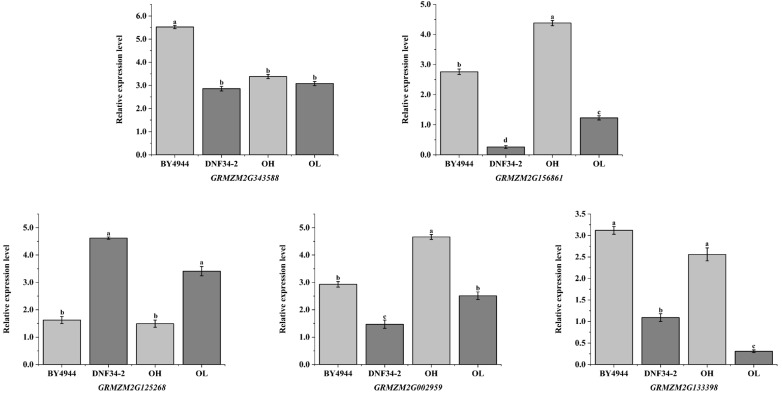
Validation of candidate genes by RT-qPCR. The x-axis represents the two parental groups, the high-oil group (OH) and low-oil group (OL), while the y-axis indicates the relative gene expression levels. Lowercase letters above the bars denote the statistical significance of intergroup differences: groups sharing the same letter show no significant difference, whereas groups with different letters exhibit statistically significant differences (*p* < 0.05).

Utilizing maize *ACT* (J01238.1) as the internal reference gene, the expression patterns of candidate genes were examined in OH, OL, and parental lines. As depicted in Figure 6, the five genes displayed distinct expression levels across the samples, yet their expression trends were consistent with the transcriptome sequencing results, thereby confirming the reliability of the screening outcomes. GRMZM2G156861 and GRMZM2G343588 exhibited negative regulatory roles in maize oil content, while GRMZM2G133398, GRMZM2G125268, and GRMZM2G002959 functioned as positive regulators of this trait.

## 3. Discussion

### 3.1. Utilizing Genetic Modeling in Silage Maize Breeding

Conventional breeding methods have consistently proven to be labor-intensive and cost-prohibitive. However, the utilization of doubled haploid (DH) populations demonstrates significant advantages in mitigating complexities associated with heterozygous loci through dominance and non-epistatic effects, thereby minimizing environmental variance interference [17]. Concurrently, DH-based breeding technology accelerates the attainment of homozygosity and substantially reduces the duration of the breeding cycle [18].

In this investigation, we examined the phenotypic variation and genetic architecture of oil content traits by constructing a doubled haploid (DH) population comprising 274 lines derived from two distinctly divergent parental inbred lines, BY4944 and DNF34-2. Our analysis revealed that the offspring oil content traits exhibited a normal distribution pattern, which is highly advantageous for quantitative trait loci (QTL) mapping.

### 3.2. High-Density Genetic Mapping and QTL Analysis

The construction of a genetic linkage map is a foundational prerequisite for deciphering the maize genome and identifying genes that govern oil content-related traits [18]. To ensure the precision of genetic mapping, we implemented a rigorous screening of progeny sequences through Genotyping-by-Targeted Sequencing (GBTS) to obtain high-fidelity molecular markers.

Ultimately, 5400 markers were obtained that were distributed among ten linkage groups, with the average interval between adjacent markers ranging from 0.49 cM to 0.95 cM, indicating high linkage map resolution. Nonetheless, notable disparities in genetic distances and considerable variability in numbers of molecular markers within the linkage groups were observed. This phenomenon stemmed from the heterogeneous distribution of SNP molecules across the chromosomes of the parental lines, culminating in variations in the reassortment rates among the hybrid offspring, thereby leading to divergent marker counts within each linked group [19].

Subsequent QTL analysis of the high-density genetic maps in conjunction with phenotypic data detected a total of 13 QTLs that were intricately linked to oil content traits that collectively accounted for phenotypic variations ranging from 0.01% to 5.06%. Notably, qOIL-1-1, situated on chromosome 1 within the region spanning 240.93 Mb to 256.57 Mb, emerged as a pivotal candidate. With a substantial LOD value of 3.34, qOIL-1-1 explained 5.06% of the phenotypic variation in oil content, the largest effect observed, thereby signifying its important role as a main effect QTL and highlighting its potential applicability for marker-assisted selection strategies.

Previous studies have demonstrated the presence of oil content-regulating genes across all 10 maize chromosomes [6,7,8,9], prompting comparisons between these QTLs and those identified in the current study. For example, Yang et al. [6] identified 42 maize QTLs governing fatty acid composition and grain oil content across all 10 chromosomes, including notable QTLs such as lin4 (PVE: 4%) on chromosome 4, which is close to qOIL-4-1 identified herein, and ste6-2 (PVE: 9.3%) on chromosome 6, which is close to qOIL-6-1 identified herein. Additionally, QTLs identified by Yang et al. [9] on chromosomes 1, 4, 5, 6, 7, 8, and 10 included qOIL06-02 (PVE: 6.4%) on chromosome 6, which is relatively close to (and possibly identical to) qOIL-6-1 identified herein. Moreover, Yu et al. [10] identified nine unconditional QTLs on chromosomes 1, 2, 4, 5, 6, 8, 9, and 10 associated with oil content, including oilc4-1 (PVE: 2.6%) located on chromosome 4 close to (and possibly identical to) qOIL-4-3 identified in this study. It is noteworthy that although oilc4-1 is associated with oil content and is located close to qOIL-4-2, it is conditioned by starch content. These regions within chromosome 4 evidently exert substantial influence on oil content regulation, as evidenced by the consistent detection of stable QTLs across diverse genetic backgrounds. Such insights hold promise for fine QTL mapping endeavors and candidate gene identification in subsequent investigations. Furthermore, it is plausible that unreported QTLs might exhibit population-specific effects influenced by distinct genetic backgrounds [20].

The novel QTLs identified in this investigation complement prior research findings. Notably, qOIL-4-2 overlaps with qOIL-4-3, and qOIL-8-1 overlaps with qOIL-8-2, signifying potential interplay among QTLs within close chromosomal proximity. This phenomenon of clustered distribution underscores the multifaceted nature of QTL localization, wherein multiple traits may be influenced by one or more QTLs situated in parallel or analogous locations [21]. Such observations enrich our understanding of the complex genetic architecture governing maize oil content and pave the way for elucidating intricate regulatory mechanisms underlying this economically significant trait.

### 3.3. Screening of Candidate Genes

This study conducted GO enrichment analysis on differentially expressed genes related to oil content, identifying GO terms directly associated with lipid biosynthesis and metabolism, as well as the indirect functional roles of oil-associated genes. Among the biological process and molecular function categories, the most enriched subterms were RNA biosynthetic process and ligase activity, respectively. Previous research has demonstrated that lipids can directly regulate RNA activity in a simplified synthetic system [22] and modulate ubiquitin ligase activity [23]. Our findings indicate that oil-related differentially expressed genes have regulatory impacts on RNA biosynthetic processes and ligase activity at the transcriptional level. Specifically, α-1,2-mannosyltransferase plays a role in lipid-linked oligosaccharide assembly [24], while geranylgeranyl transferase requires knockdown to activate key regulators of membrane protein endocytic transport and subcellular trafficking, a method commonly used to investigate lipid homeostasis [25]. These results suggest that oil-regulatory genes influence enzymatic activities, warranting further investigation to validate their mechanistic coupling with lipid functionality. KEGG pathway analysis further delineated the biological roles and metabolic pathways of oil content-associated genes, including direct involvement in ether lipid metabolism [26], diterpenoid biosynthesis [27], vitamin B6 metabolism [28], linoleic acid metabolism [29], steroid biosynthesis [30], α-linolenic acid metabolism [31], citrate cycle (TCA cycle) [32], pantothenate and CoA biosynthesis [33], glycerophospholipid metabolism [34], and propanoate metabolism [35]. Moreover, although previous studies have indicated positive correlations between maize kernel oil content and levels of protein, tryptophan, and lysine, our data reveal a significant enrichment of oil-related genes within the metabolic pathways of valine, leucine, isoleucine, serine, threonine, and glycine.

We identified five candidate genes that regulate the oil content in maize. GRMZM2G133398, homologous to Arabidopsis AT3G24650 (annotated as an AP2/B3-like transcription factor family protein, encoding ABA-INSENSITIVE 3 [ABI3]), was previously believed to influence lipid accumulation indirectly via FUSCA3 (FUS3). However, Zheng et al. [36] demonstrated that ABI3 independently activates lipid biosynthesis. GRMZM2G125268, homologous to Arabidopsis AT3G48000 (which encodes mitochondrial aldehyde dehydrogenase RF2B), oxidizes short-chain fatty aldehydes to produce fatty acids with one fewer carbon atom, as reported by Liu et al. [37] in maize. GRMZM2G343588, homologous to Arabidopsis AT3G16785 (which encodes phospholipase D1), catalyzes the conversion of diacylglycerol (DAG) to triacylglycerols (TAG) [38,39]. These three genes are believed to act as positive regulators of oil accumulation. In contrast, GRMZM2G156861, homologous to Arabidopsis AT1G55020 (encoding lipoxygenase 1 [LOX1]), promotes fatty acid oxidation and alters lipid metabolism [40], while GRMZM2G002959, homologous to Arabidopsis AT3G51840 (encoding acyl-CoA oxidase 4), catabolizes long-chain fatty acids [41]. These two genes may act as negative regulators during oil biosynthesis.

The mechanistic roles of these candidate genes in maize lipid synthesis and metabolism have yet to be fully elucidated. Future studies should prioritize comprehensive screening and validation of target genes using advanced methodologies, including genome-wide association analysis, QTL fine mapping, and molecular marker-assisted breeding. Systematic functional characterization—such as CRISPR-based gene editing, lipidomic profiling, and spatiotemporal expression analysis—will be crucial to unravel their regulatory networks and interactions within lipid metabolic pathways. This work lays the foundation for refining genetic strategies to optimize the oil content in maize through precision breeding.

## 4. Materials and Methods

### 4.1. Plant Material

A total of 274 DH lines were used in this study. These lines were derived from BY4944 and DNF34-2 parents known for their significant differences in oil content traits. Cultivation took place during the 2022 growing season at the National Modern Agriculture Demonstration Park of Heilongjiang Academy of Agricultural Sciences, located in Harbin, China (longitude:126.53°, latitude:45.80°). The experimental field followed a two-row planting configuration, with a plant spacing of 15 cm and a row spacing of 60 cm, adhering to standard agronomic practices. General field management techniques were applied throughout the growing period. For genetic analysis, young leaves from plants in the seedling stage (v3 stage) were placed in liquid nitrogen and stored at −80 °C until further analysis.

### 4.2. Measurement of Oil Content

At physiological maturity (45 days after pollination), 10 plants each from parental lines and 274 DH lines were randomly selected for ear harvesting, with three biological replicates. The labeled samples were dried in mesh bags and threshed after complete desiccation, with maize kernels subsequently employed for oil content determination.

Quantitative analysis of kernel oil content was performed using the FOSS NIRS system (Infratec™ 1241) through near-infrared spectroscopy scanning (spectral range 950–1650 nm). The spectral acquisition program (Scan mode) was activated to collect 32 spectral scans per sample, with data archived in CAL format.

### 4.3. Sequencing and Genotyping

Genomic DNA was extracted using the CTAB method [42] to minimize DNA degradation and contamination, as confirmed by 1% agarose gel electrophoresis. Subsequently, the purity and concentrations of the DNA samples were assessed using a NanoDrop UV-VIS spectrophotometer and Qubit 3.0 fluorometer, respectively. High-quality DNA samples were used for the construction of targeted sequencing libraries. Genotyping by target sequencing (GBTS) was employed to generate sequences for analysis, with sequencing performed on the MGI2000 platform using PE150 mode [43]. Raw reads underwent quality control by using Fastp software (version 0.20.0, parameters: -n10-q20-u40), which involved the removal of adapter sequences and paired reads containing more than 10 ‘N’ bases or low-quality (Q ≤ 20) bases exceeding >40% of the read length. The remaining clean reads were aligned to the reference genome sequences (Zm-B73-REFERENCE-NAM-5.0) using Burrows-Wheeler Aligner (BWA-MEM) software (version v0.7.17). Following alignment, statistical analysis was conducted to detect parental marker deletions and heterozygosity; then, aa × bb markers were screened for further analysis. SNPs with a missing rate >10%, a minor allele frequency (MAF) <0.05, and a sample missing rate >10% were filtered out.

### 4.4. Construction, Evaluation, and QTL Mapping of Genetic Linkage Map

To construct the genetic linkage map, we utilized the mstmap function from the R package ASMap [44], employing a stringent significance threshold (parameter: P.UE = 1 × 10^−6^). Collinearity analysis between the physical location and genetic distance of each marker was performed to ensure map accuracy.

For the QTL mapping of oil content-related traits, we employed the composite interval mapping (CIM) method using the cim() function of R/qtl using the parameter settings of 1000 permutations and a significance level of >2.5. The QTLs with the highest contribution rates were classified as main effect QTLs. QTLs were named according to a standardized convention, starting with the letter “*q*” followed by the trait abbreviation, chromosome number, and QTL serial number.

### 4.5. RNA Sequencing (RNA-Seq) Analysis and RT-qPCR

Thirty high-oil (OH) and thirty low-oil (OL) plants were selected. Ten randomly selected plants per phenotype were pooled to form three biological replicates. At the full maturity stage, kernels were collected from individual maize plants, immediately flash-frozen in liquid nitrogen, and subsequently stored at −80 °C freezer for preservation until further analysis. The modified RNA extraction method for mature maize kernels described by Zhang et al. [45] was employed in this study. RNA quality assessment, library preparation, and sequencing were conducted on the Illumina NovaSeq. 6000 sequencing platform, performed by Guangzhou Genedenovo Biotechnology Co., Ltd, Guangzhou, China. The resulting reads were filtered by HISAT (v.2.0.4) with default parameters and subsequently aligned to the maize reference genome (Zm-B73-REFERENCE-NAM-5.0). Data quality control and the read counts were sequentially performed using FastQC (v.0.11.9) and FeatureCounts (v.2.0.1). The differentially expressed genes (DEGs) between the two samples were identified based on the following criteria: fold-change >1.5 and false discovery rate (FDR) <0.05. The data were analyzed using the TBtools (v2.210).

Following sequencing, functional annotation of homologous proteins and comparative metabolic pathway analyses were performed using databases including Gene Ontology (GO) and Kyoto Encyclopedia of Genes and Genomes (KEGG), enabling the identification of differentially expressed genes (DEGs) associated with oil content. Subsequently, the physical coordinates of the QTL mapping intervals were aligned with the maize genome assembly in the NCBI database to delineate genomic features within these regions. Genes exhibiting significant differential expression localized within QTL-corresponding physical intervals were designated as candidate genes.

The expression patterns of candidate genes were validated by RT-qPCR analysis using SYBR Green qPCR Mix (Mona Biotech Co., Ltd., Suzhou, China), with maize *ACT* (J01238.1) (Gene ID: LOC100282267) serving as the internal reference gene. Detailed information regarding the RT-qPCR amplification system and primer sequences is provided in Supplementary Tables S2 and S3.

## 5. Conclusions

This study utilized 274 DH lines derived from BY4944 and DNF34-2 to conduct comprehensive genetic analyses, QTL detection, and candidate gene exploration aimed at elucidating the genetic architecture underlying the oil content in silage maize. Leveraging these materials, we constructed a high-density genetic linkage map comprising 5400 SNP markers, enabling the precise localization of genetic loci influencing oil content traits. Through rigorous QTL analysis, we detected a total of 13 QTLs associated with oil content. The most prominent QTL, qOIL-1-1, positioned at 240.93-256.57 Mb on chromosome 1, is potentially a main effect QTL driving oil content variation.

Furthermore, our investigation identified five candidate genes, whose expression trends were validated via RT-qPCR to align with transcriptomic data, thereby reinforcing the robustness of our findings. This study not only provides critical insights into the genetic and molecular mechanisms underlying this economically significant trait but also establishes a rigorous framework for subsequent research endeavors.

## Figures and Tables

**Table 1:** Descriptive statistical results of parental and DH line traits.

Traits	Parents					DH Lines				
	Maternal Parent	Male Parent	*T* Value	Median Value	*K*-*S* Value	Sig Value	Mean	Standard Deviation	Environmental Variance	Coefficient of Variation
Oil content/%	9.58	3.41	−34.38 **	6.49	0.034	0.200	5.72	1.07	0.14	18.25

**: *p* < 0.05.

**Table 2:** Basic characteristics of maize genetic map constructed by DH population.

Linkage Group	Marker Number	Genetic Distance/cM	Average Map Distance/cM	Maximum Interval/cM
LG01	1014	627.74	0.62	27.71
LG02	609	466.70	0.77	33.42
LG03	737	524.61	0.71	42.76
LG04	495	344.61	0.70	27.22
LG05	573	283.57	0.49	19.55
LG06	388	369.12	0.95	41.30
LG07	480	353.16	0.74	39.37
LG08	474	378.41	0.80	40.02
LG09	328	260.69	0.79	39.37
LG10	302	255.91	0.85	13.06
ALL	5400	3864.51	0.74	42.76

**Table 3:** QTL mapping of maize oil content.

Chromosome	QTL	Peak/cM	Physical Interval/Mb	LOD Value	Contribution Rate/%	Additive Effect
1	qOIL-1-1	100.01	240.93~256.57	3.34	5.06	−0.25~−0.22
3	qOIL-3-1	203.68	6.53~12.14	3.03	2.78	−0.36~−0.32
	qOIL-3-2	243.70	160.12~170.98	4.72	0.02	−0.41~−0.40
	qOIL-3-3	524.61	169.01~178.32	2.73	1.06	−0.43~−0.41
4	qOIL-4-1	46.48	11.40~139.47	4.71	0.05	−0.43~−0.33
	qOIL-4-2	64.25	150.66~166.04	5.15	1.89	−0.37~−0.34
	qOIL-4-3	76.11	157.95~177.07	3.65	0.01	−0.36~−0.32
5	qOIL-5-1	20.78	3.82~7.25	3.37	3.52	−0.31~−0.28
	qOIL-5-2	283.58	15.21~27.66	2.59	1.11	−0.23~−0.22
6	qOIL-6-1	155.34	88.91~102.89	3.51	0.47	−0.40~−0.34
	qOIL-6-2	186.57	109.48~130.94	3.22	0.48	−0.36~−0.26
8	qOIL-8-1	300.00	22.65~116.15	3.06	0.10	−0.32~−0.28
	qOIL-8-2	311.27	25.34~141.13	3.00	0.01	−0.33~−0.28

**Table 4:** Details of candidate genes related to maize oil content.

Chr.	QTL	Gene ID	Homologous Genes in Arabidopsis	Functional Annotation
3	qOIL-3-2	GRMZM2G133398	AT3G24650	Regulatory protein viviparous-1 [*Zea mays*]
3	qOIL-3-2	GRMZM2G156861	AT1G55020	lipoxygenase [*Zea mays*]
4	qOIL-4-2	GRMZM2G125268	AT3G48000	mitochondrial aldehyde dehydrogenase RF2B [*Zea mays*]
6	qOIL-6-2	GRMZM2G002959	AT3G51840	acyl-CoA oxidase 4 [*Zea mays*]
8	qOIL-8-1	GRMZM2G343588	AT3G16785	phospholipase D16 [*Zea mays*]

## Data Availability

We ensure that the permissions of the *Zea mays* listed in the manuscript were obtained. All data generated or analyzed during this study are included in this published article and its Supplementary Information Files.

## Supplementary Materials

The following supporting information can be downloaded at https://www.mdpi.com/article/10.3390/plants14081181/s1, Figure S1: The distribution of markers on the genome; Figure S2: Collinear graph; Figure S3: Correlation coefficient heatmap; Table S1: Sequencing data and filtering of parental and DH line; Table S2: Gene name and primer sequence; Table S3: RT-qPCR amplification system.

## Abbreviations

The following abbreviations are used in this manuscript:

QTL	quantitative trait loci
DH	double haploid complex interval mapping (CIM)
SNP	single nucleotide polymorphism
LOD	logarithm of odds
TAG	triacylglycerol
PPP	pentose phosphate pathway
AIC	Akaike information criterion
GBTS	genotyping by target sequencing
BWA	Burrows–Wheeler aligner
GO	Gene Ontology
KEGG	Kyoto Encyclopedia of Genes and Genomes
K-S	Kolmogorov–Smirnov
BP	biological process
CC	cell component
MF	molecular function
ABI3	ABA-INSENSITIVE 3
KASI-β	ketoacyl-[acyl carrier protein] synthase I
BRs	brassinosteroids
PVE	phenotypic variation explained
ABI3	ABA-INSENSITIVE 3
DAG	diacylglycerol
FUS3	FUSCA3
