# Supplementary material for: QTL Mapping and Candidate Gene Screening for Enhancing Oil Content in Silage Maize
Source: Plants (Basel). 2025 Apr 10;14(8):1181. doi: 10.3390/plants14081181 (PMC12030292; doi:10.3390/plants14081181)
Supplement: Supplementary file 1 [file plants-14-01181-s001.zip › Supplementary Table 1.pdf]

Table S1 Sequencing data and filtering of parental and DH line

Sample	Raw Reads Number	Clean Reads Number	Raw Bases(Mb)	Clean Bases(Mb)	Effective Rate(%)	Q20(%)	Q30(%)	Coverage(%)
22hd27979	1915766	1915766	287.36	256.65	89.31	98.97	97.03	88.61
22hd27946	2861388	2861388	429.21	402.65	93.81	98.75	96.38	91.24
22hd27914_1	3236658	3236658	485.50	442.17	91.07	98.86	96.69	91.06
22hd27960_1	3583680	3583680	537.55	494.37	91.97	98.87	96.73	90.62
22hd27815_1	3657430	3657430	548.61	506.13	92.26	98.73	96.31	90.79
22hd27880_2	3703046	3703046	555.46	513.78	92.5	98.87	96.7	90.19
22hd27854_1	3765992	3765992	564.90	525.60	93.04	98.82	96.59	90.89
22hd28130	2192116	2192116	328.82	299.44	91.06	98.8	96.48	89.21
22hd28207	2321106	2321106	348.17	314.08	90.21	99.01	97.15	88.87
22hd27856_1	3118986	3118986	467.85	435.14	93.01	98.82	96.62	89.81
22hd27881_1	3448534	3448534	517.28	475.67	91.96	98.82	96.59	90.03
22hd27867_1	3365250	3365250	504.79	464.37	91.99	98.83	96.62	89.88
22hd27852_2	3852940	3852940	577.94	531.17	91.91	98.74	96.35	90.45
22hd27855	4046330	4046330	606.95	560.27	92.31	98.73	96.32	90.54
22hd27950_1	1317394	1317394	197.61	184.46	93.35	98.67	96.12	90.16
22hd27983_1	3067554	3067554	460.13	418.28	90.91	98.94	96.92	91.53
22hd27959_1	3057146	3057146	458.57	424.71	92.62	98.67	96.11	90.41
22hd28018	2946528	2946528	441.98	390.38	88.33	99.02	97.16	90.25
22hd28048_1	3412478	3412476	511.87	451.47	88.2	99.09	97.37	90.81
22hd28196	3466478	3466478	519.97	457.58	88	98.94	96.88	88.97
22hd28140_1	3239732	3239732	485.96	442.81	91.12	98.94	96.92	90.53
22hd27953_1	3503654	3503654	525.55	484.26	92.14	98.82	96.61	89.50
22hd27973_1	3464380	3464380	519.66	476.45	91.69	98.84	96.62	91.03
22hd27980_1	3668846	3668846	550.33	504.11	91.6	98.88	96.75	90.13
22hd27882	3585600	3585600	537.84	497.35	92.47	98.76	96.39	90.91
22hd27849_1	3702592	3702592	555.39	512.73	92.32	98.87	96.74	90.39
22hd27939_1	3256550	3256550	488.48	438.68	89.8	98.97	96.99	91.28
22hd27839_1	3118666	3118664	467.80	427.06	91.29	98.91	96.87	90.25
22hd28195	3531802	3531802	529.77	487.91	92.1	98.87	96.74	89.73
22hd27861_1	2327438	2327438	349.12	326.91	93.64	98.58	95.86	90.48
22hd28099	3284906	3284906	492.74	451.45	91.62	98.9	96.78	91.66
22hd28111_1	3016570	3016570	452.49	401.97	88.84	98.98	97.04	89.45
22hd28068_1	3425628	3425628	513.84	464.51	90.4	98.92	96.85	90.42
22hd28138_1	3094516	3094516	464.18	421.80	90.87	98.91	96.83	89.87
22hd27819	3278910	3278910	491.84	461.56	93.84	98.62	96	90.53
22hd27866_1	3662878	3662878	549.43	514.99	93.73	98.74	96.35	90.58
22hd28052_1	2297800	2297800	344.67	312.32	90.61	98.97	97.05	90.15
22hd28017_1	2503860	2503860	375.58	332.39	88.5	99.06	97.3	89.93
22hd28210_1	3043882	3043882	456.58	406.84	89.11	98.89	96.77	88.54
22hd27844_1	2913646	2913646	437.05	402.08	92	98.77	96.4	90.22

22hd27822_1	3519126	3519126	527.87	475.01	89.99	98.93	96.9	89.97
22hd28104	3672550	3672550	550.88	501.12	90.97	98.89	96.75	91.94
22hd27993	2141214	2141214	321.18	284.46	88.57	98.97	97	90.24
22hd28077	2166218	2166218	324.93	280.68	86.38	99.11	97.43	88.82
22hd28053_1	2865448	2865448	429.82	381.12	88.67	99.02	97.15	88.63
22hd27847_1	3259942	3259942	488.99	452.39	92.51	98.79	96.49	89.46
22hd28006_1	3141262	3141262	471.19	426.17	90.45	98.86	96.68	90.32
22hd27812	3609734	3609734	541.46	499.88	92.32	98.8	96.52	89.95
22hd28193	2697648	2697648	404.65	364.05	89.97	98.96	96.99	90.88
22hd27840_1	3659120	3659120	548.87	507.75	92.51	98.89	96.82	90.25
22hd27948_1	3053418	3053418	458.01	424.15	92.61	98.68	96.15	90.24
22hd27995_1	3547486	3547486	532.12	486.39	91.41	99	97.14	90.70
22hd28060_1	3681862	3681862	552.28	506.23	91.66	98.84	96.63	90.86
22hd28019_1	3609544	3609544	541.43	490.29	90.55	98.9	96.79	91.32
22hd27932_1	3316002	3316002	497.40	459.08	92.3	98.77	96.4	89.62
22hd28091_1	3928678	3928678	589.30	537.69	91.24	98.97	97.04	91.15
22hd27879_1	3743498	3743498	561.52	517.14	92.1	98.79	96.49	90.64
22hd27827_1	4200298	4200298	630.04	584.40	92.75	98.8	96.53	90.44
22hd28031_1	4507618	4507618	676.14	623.49	92.21	98.75	96.37	91.48
22hd28182_1	4159988	4159988	624.00	571.37	91.57	98.84	96.64	90.36
22hd27976_1	5149154	5149154	772.37	706.31	91.45	98.9	96.84	90.18
22hd27825_1	4555250	4555250	683.29	630.61	92.29	98.75	96.34	89.28
22hd28088_1	939620	939620	140.94	127.14	90.21	98.96	96.99	87.40
22hd27913_1	2523988	2523988	378.60	349.94	92.43	98.78	96.45	91.05
22hd28090	2988322	2988322	448.25	398.25	88.84	99	97.12	91.23
22hd27996_1	3110176	3110176	466.53	432.41	92.69	98.8	96.54	91.39
22hd27991_1	3224598	3224598	483.69	431.64	89.24	98.86	96.66	90.47
22hd28121_1	3711574	3711574	556.74	504.88	90.69	98.97	97.05	89.91
22hd27904_1	3982282	3982282	597.34	554.21	92.78	98.71	96.25	90.92
22hd28058	3979710	3979710	596.96	549.83	92.11	98.86	96.69	91.17
22hd27909_1	4044246	4044246	606.64	560.01	92.31	98.84	96.65	90.46
22hd27888_1	4037142	4037142	605.57	560.32	92.53	98.82	96.57	90.24
22hd28054	3868578	3868576	580.29	522.43	90.03	98.98	97.01	90.57
22hd27911_1	2422560	2422560	363.38	338.03	93.02	98.78	96.5	90.25
22hd27951_1	2822716	2822716	423.41	388.85	91.84	98.77	96.42	91.24
22hd28069_1	3214942	3214942	482.24	438.76	90.98	98.89	96.77	91.86
22hd27901_1	3279502	3279502	491.93	451.39	91.76	98.74	96.33	89.81
22hd29782_1	3225568	3225568	483.84	436.17	90.15	98.92	96.88	90.24
22hd27969_1	3285506	3285506	492.83	452.23	91.76	98.8	96.49	90.63
22hd27851_1	4244944	4244942	636.74	592.35	93.03	98.77	96.44	90.32
22hd28101	401676	401676	60.25	54.37	90.24	98.83	96.58	82.35
22hd28153_1	2021608	2021608	303.24	269.48	88.87	98.9	96.79	87.30
22hd28107	1902560	1902560	285.38	261.84	91.75	98.82	96.57	90.08
22hd28146	3873702	3873702	581.06	536.32	92.3	98.82	96.58	89.46

22hd28050_1	985098	985098	147.76	136.36	92.28	98.76	96.39	87.98
22hd27841_2	2375886	2375884	356.38	313.99	88.11	98.92	96.85	89.58
22hd28144	2936768	2936768	440.52	396.81	90.08	99.04	97.24	90.22
22hd28112_1	3227166	3227166	484.07	434.83	89.83	98.94	96.91	90.29
22hd28095_1	3793976	3793976	569.10	517.22	90.88	98.83	96.59	90.83
22hd27940_1	2581980	2581980	387.30	360.29	93.03	98.62	95.99	90.47
22hd27886_1	2621346	2621346	393.20	371.05	94.36	98.55	95.78	89.78
22hd27992	3149240	3149240	472.39	418.73	88.64	99.02	97.14	89.32
22hd28188	3423836	3423836	513.58	456.68	88.92	98.98	97.06	89.95
22hd27818_1	3581646	3581646	537.25	498.13	92.72	98.84	96.66	89.93
22hd27843_1	3691092	3691092	553.66	512.27	92.52	98.79	96.52	90.60
22hd28108	3907448	3907448	586.12	525.74	89.7	99.08	97.36	91.29
22hd27934_1	2934042	2934042	440.11	404.28	91.86	98.73	96.28	89.94
22hd28203	2269900	2269900	340.49	309.62	90.94	98.88	96.74	88.91
22hd27883_1	3183568	3183568	477.54	442.31	92.62	98.82	96.59	90.57
22hd27964_1	3177976	3177976	476.70	434.87	91.22	98.94	96.93	90.36
22hd27921_1	3467992	3467992	520.20	485.93	93.41	98.67	96.13	90.52
22hd27814_2	3430494	3430494	514.57	469.15	91.17	98.91	96.84	89.18
22hd28206	3500252	3500252	525.04	475.15	90.5	98.92	96.86	89.81
22hd27920_1	2993346	2993346	449.00	411.29	91.6	98.82	96.57	90.39
22hd28097_2	2908022	2908022	436.20	400.15	91.73	98.94	96.96	90.23
22hd28046_1	3103438	3103438	465.52	404.54	86.9	99.09	97.37	90.00
22hd28027_1	3577154	3577154	536.57	478.63	89.2	98.98	97.04	91.15
22hd27912_1	3171208	3171208	475.68	438.10	92.1	98.78	96.46	91.09
22hd28005_1	3134166	3134166	470.12	425.43	90.49	98.98	97.04	90.63
22hd28059_1	2642210	2642210	396.33	358.64	90.49	98.88	96.74	89.53
22hd28194	3374848	3374848	506.23	462.88	91.44	98.89	96.81	89.36
22hd28205	1049186	1049186	157.38	141.17	89.7	98.99	97.06	84.18
22hd28141_2	3857426	3857426	578.61	521.95	90.21	98.91	96.83	90.13
22hd28136_1	2341186	2341186	351.18	319.41	90.95	98.92	96.87	88.88
22hd27981_1	2524522	2524522	378.68	343.98	90.84	98.81	96.54	89.95
22hd28098_1	2593940	2593940	389.09	349.38	89.79	98.97	97.02	90.93
22hd27919_1	2748058	2748058	412.21	382.41	92.77	98.71	96.23	90.23
22hd27823_1	3469144	3469144	520.37	479.40	92.13	98.69	96.16	89.64
22hd27918_1	3483676	3483676	522.55	476.53	91.19	98.87	96.71	91.17
22hd27846	3519428	3519428	527.91	493.56	93.49	98.67	96.12	90.15
22hd28129	3504502	3504502	525.68	483.44	91.97	98.86	96.71	90.88
22hd28014	3690982	3690982	553.65	498.44	90.03	98.95	96.96	90.54
22hd28055_1	3755804	3755804	563.37	503.14	89.31	99.07	97.32	90.67
22hd28150	3711658	3711658	556.75	515.94	92.67	98.72	96.24	91.09
22hd28094_1	4014980	4014980	602.25	542.11	90.02	99.01	97.13	91.66
22hd27876_1	4036520	4036520	605.48	566.28	93.53	98.79	96.51	90.19
22hd28021_1	3952764	3952764	592.91	510.80	86.15	99.08	97.33	90.18
22hd27916_1	2598756	2598756	389.81	361.73	92.8	98.83	96.62	90.63

22hd28163	3504184	3504184	525.63	468.17	89.07	99.05	97.26	90.21
22hd27917_1	2273172	2273172	340.98	318.84	93.51	98.65	96.06	91.08
22hd27835_1	3159298	3159298	473.89	439.77	92.8	98.77	96.45	90.34
22hd27972_1	3472496	3472496	520.87	482.01	92.54	98.68	96.11	91.05
22hd27997_1	3664836	3664836	549.73	495.03	90.05	98.91	96.83	90.39
22hd28137_1	3304324	3304324	495.65	450.77	90.95	98.83	96.59	90.38
22hd27862_1	2439724	2439724	365.96	344.40	94.11	98.62	96.01	90.61
22hd27947_1	3337944	3337944	500.69	466.32	93.14	98.61	95.93	91.14
22hd28202	3886750	3886750	583.01	529.72	90.86	98.83	96.59	89.94
22hd28020_1	4491472	4491472	673.72	612.50	90.91	98.89	96.78	91.07
22hd27985_1	3241832	3241832	486.27	435.06	89.47	98.98	97.03	91.16
22hd28057	2982344	2982344	447.35	400.42	89.51	98.89	96.74	91.21
22hd27858_1	2396810	2396808	359.52	334.51	93.04	98.78	96.5	90.21
22hd28139	3087462	3087462	463.12	418.67	90.4	98.97	97.04	90.06
22hd27958_1	2782384	2782384	417.36	364.93	87.44	99.06	97.27	90.33
22hd27816	3756062	3756062	563.41	527.46	93.62	98.72	96.3	90.05
22hd27965	2840956	2840956	426.14	387.59	90.95	98.85	96.66	90.55
22hd27978_1	3282864	3282864	492.43	453.12	92.02	98.89	96.78	90.14
22hd28022_1	4315718	4315718	647.36	595.93	92.06	98.92	96.89	91.65
22hd28013_1	4104966	4104966	615.74	556.99	90.46	98.92	96.87	90.74
22hd28089_1	1538070	1538070	230.71	211.77	91.79	98.83	96.62	89.98
22hd27924_1	4478636	4478636	671.80	616.47	91.77	98.95	96.99	90.69
22hd27871_1	3520936	3520936	528.14	488.21	92.44	98.83	96.64	90.17
22hd27820_3	2984930	2984930	447.74	415.95	92.9	98.71	96.24	89.37
22hd28030_1	2621752	2621752	393.26	347.52	88.37	99.05	97.28	90.78
22hd28117_1	4035604	4035604	605.34	554.31	91.57	98.91	96.84	91.67
22hd27957_1	2989670	2989668	448.45	414.16	92.36	98.75	96.36	89.79
22hd28181	3195258	3195258	479.29	432.65	90.27	98.89	96.75	89.40
22hd28176	1763262	1763262	264.49	228.70	86.47	99.13	97.44	87.46
22hd27949_1	2101778	2101776	315.27	291.07	92.33	98.75	96.37	90.32
22hd28149	3093604	3093604	464.04	417.15	89.9	98.96	96.98	89.94
22hd27826_1	3933142	3933142	589.97	542.79	92	98.87	96.73	89.72
22hd27884_1	3830842	3830842	574.63	533.47	92.84	98.72	96.27	90.69
22hd27966_1	2486044	2486044	372.91	336.44	90.22	98.96	97	89.69
22hd27860_1	3768796	3768796	565.32	523.88	92.67	98.74	96.33	91.08
22hd27875_1	1068454	1054158	160.27	149.41	93.23	97.91	93.74	91.23
22hd27933_1	3182694	3147038	477.40	445.27	93.27	97.82	93.49	89.50
22hd28029_1	2017338	2001038	302.60	278.97	92.19	97.73	93.33	89.74
22hd28080_1	2557872	2534942	383.68	352.16	91.78	97.94	93.83	90.06
22hd28160	2649892	2625404	397.48	370.68	93.26	97.68	93.2	88.79
22hd27900_1	2836864	2802326	425.53	396.84	93.26	97.55	92.93	90.89
22hd27905_1	2959876	2928420	443.98	421.56	94.95	97.64	93.04	91.42
22hd28105_1	1931088	1912254	289.66	273.56	94.44	97.35	92.48	89.42
22hd28035_1	3157258	3123732	473.59	435.87	92.03	97.84	93.58	89.79

22hd27961	3073330	3046708	461.00	414.73	89.96	97.9	93.75	89.29
22hd28070_1	3486262	3450030	522.94	486.37	93.01	97.86	93.58	88.96
22hd27941_1	2723696	2693714	408.55	385.03	94.24	97.7	93.2	90.69
22hd27890_1	3200502	3170472	480.08	446.78	93.07	97.71	93.21	90.44
22hd27928_1	3252818	3223280	487.92	456.81	93.62	97.78	93.39	90.91
22hd27891_1	3207994	3178636	481.20	448.58	93.22	97.74	93.33	90.89
22hd28087_1	2570914	2540558	385.64	365.92	94.89	97.24	92.23	90.05
22hd27896_1	3000512	2963048	450.08	426.79	94.83	97.51	92.79	89.89
22hd28038_1	3046824	3018002	457.02	428.33	93.72	97.86	93.65	89.07
22hd27863_1	2801402	2775884	420.21	394.99	94	97.64	93.08	89.85
22hd27870_1	1915924	1888880	287.39	268.51	93.43	97.63	93.05	89.14
22hd27872_1	2514344	2488478	377.15	353.56	93.74	97.71	93.23	90.29
22hd28164_1	2452538	2429674	367.88	345.26	93.85	97.74	93.32	88.30
22hd27868_1	2814830	2746560	422.22	391.71	92.77	97.77	93.42	89.28
22hd28036	2704734	2674626	405.71	378.83	93.37	97.62	92.99	90.65
22hd27906	2748766	2724952	412.31	376.42	91.29	97.81	93.48	89.92
22hd28109	2173952	2152626	326.09	310.64	95.26	97.44	92.61	89.41
22hd28135_1	2369632	2352836	355.44	336.08	94.55	97.72	93.24	89.35
22hd27838_1	2898704	2862900	434.81	402.17	92.5	97.59	93.02	90.11
22hd27898_1	2777494	2746630	416.62	383.33	92.01	97.8	93.49	89.53
22hd28003_1	3053468	3015794	458.02	418.67	91.41	97.8	93.51	89.73
22hd28134	3027364	2999330	454.10	429.02	94.48	97.53	92.89	89.37
22hd28106_2	1870922	1857990	280.64	265.47	94.6	97.65	93.08	90.05
22hd27956_1	1868026	1843270	280.20	259.82	92.73	97.78	93.49	89.49
22hd28124_1	2564976	2538170	384.75	364.53	94.75	97.54	92.9	90.15
22hd27833_1	2552156	2523644	382.82	360.26	94.11	97.58	92.94	90.33
22hd27821_1	2626362	2596936	393.95	367.86	93.38	97.67	93.16	91.00
22hd28173	2586894	2561170	388.03	368.55	94.98	97.49	92.76	89.51
22hd28122_1	2661290	2632472	399.19	381.00	95.44	97.65	93.12	90.27
22hd27864_2	2630532	2601666	394.58	374.76	94.98	97.63	93.1	90.31
22hd27837_1	2348244	2322562	352.24	333.48	94.67	97.61	93.03	91.05
22hd27999_1	3096396	3054994	464.46	423.53	91.19	97.91	93.76	89.45
22hd28042_1	2730112	2695154	409.52	381.70	93.21	97.34	92.44	89.42
22hd28011_1	3147038	3112774	472.06	440.39	93.29	97.78	93.42	88.28
22hd28116_1	2224570	2205548	333.69	316.14	94.74	97.85	93.58	90.53
22hd28007	3033194	3002848	454.98	426.76	93.8	97.62	93.05	90.33
22hd27935_1	2955612	2922760	443.34	412.14	92.96	97.71	93.27	90.35
22hd27971	3608452	3573386	541.27	504.74	93.25	97.86	93.59	90.01
22hd28045	2478626	2458282	371.79	349.22	93.93	97.83	93.55	90.68
22hd28037_1	2281942	2259946	342.29	318.65	93.09	97.9	93.7	89.09
22hd28127_1	2193200	2172964	328.98	309.63	94.12	97.5	92.76	89.61
22hd27893_1	2827136	2797604	424.07	393.29	92.74	97.57	92.94	90.12
22hd27897_1	3260706	3227966	489.11	457.01	93.44	97.99	93.93	89.98
22hd27885_1	2526266	2499510	378.94	355.72	93.87	97.73	93.33	90.68

22hd28113	2453972	2431074	368.10	350.52	95.22	97.46	92.66	89.49
22hd28024_1	2642444	2607322	396.37	373.26	94.17	97.82	93.52	90.08
22hd28043_1	2836220	2806952	425.43	396.00	93.08	97.75	93.36	88.53
22hd27937_1	2928562	2896908	439.28	384.29	87.48	97.94	93.87	89.38
22hd27892_1	2905256	2873146	435.79	406.44	93.27	97.39	92.5	90.77
22hd27857_1	2867260	2836160	430.09	395.44	91.94	97.79	93.44	89.84
22hd28161_1	2556008	2531674	383.40	362.51	94.55	97.66	93.12	89.12
22hd28008_1	2916038	2885222	437.41	402.48	92.02	97.96	93.93	89.49
22hd27926_1	2940284	2909176	441.04	399.42	90.56	97.99	93.96	90.61
22hd28082_1	2914614	2886168	437.19	397.85	91	97.8	93.51	89.79
22hd28064_1	698654	691506	104.80	98.56	94.05	97.62	93.05	90.33
22hd27829_1	2989296	2959748	448.39	420.85	93.86	97.82	93.49	90.05
22hd27927_2	3092942	3064024	463.94	434.67	93.69	97.8	93.45	91.02
22hd27925_1	3316714	3280448	497.51	471.14	94.7	97.5	92.79	91.25
22hd28166_1	2588110	2563468	388.22	367.64	94.7	97.7	93.28	89.54
22hd27936_1	2585600	2557692	387.84	364.86	94.07	97.72	93.29	91.03
22hd28147_1	2624678	2596088	393.70	375.82	95.46	97.32	92.35	89.73
22hd28125_2	1797190	1776522	269.58	249.24	92.46	97.61	93.05	88.50
22hd28065_1	2502290	2478296	375.34	350.42	93.36	97.87	93.66	89.93
22hd28155_1	2341000	2320768	351.15	326.11	92.87	97.73	93.33	89.09
22hd28086	2362200	2343300	354.33	329.97	93.13	97.82	93.53	89.57
22hd28001_1	3065490	3020454	459.82	414.61	90.17	97.89	93.75	89.73
22hd28032_1	2399304	2370844	359.90	339.28	94.27	97.56	92.94	90.04
22hd27967_1	2075310	2053432	311.30	295.11	94.8	97.66	93.16	90.40
22hd28026_1	2645742	2614072	396.86	370.16	93.27	97.44	92.68	89.30
22hd27986_1	2959220	2906532	443.88	397.76	89.61	97.75	93.47	88.28
22hd28041_1	2269044	2235252	340.36	316.23	92.91	97.63	93.1	88.92
22hd28162	830902	823088	124.64	116.59	93.55	97.71	93.29	89.03
22hd27830_1	2769864	2735366	415.48	386.85	93.11	97.44	92.64	90.43
22hd28084_1	2616622	2587588	392.49	368.25	93.82	97.53	92.9	82.02
22hd27975_1	2689046	2660178	403.36	372.25	92.29	97.85	93.61	88.09
22hd27889_1	2909790	2875096	436.47	400.45	91.75	97.58	93.02	90.97
22hd28051_1	3074554	3039010	461.18	435.41	94.41	97.49	92.8	90.99
22hd28073	2447876	2419900	367.18	343.86	93.65	97.9	93.73	89.22
22hd28133_1	2483162	2452320	372.47	350.24	94.03	97.21	92.15	87.86
22hd28000_1	3288162	3242606	493.22	455.99	92.45	97.6	93.08	90.20
22hd27970_1	2907730	2867646	436.16	398.21	91.3	97.82	93.6	88.85
22hd27832_1	3182840	3150988	477.43	447.00	93.63	97.64	93.05	90.29
22hd28063_1	3171696	3143578	475.75	444.97	93.53	97.84	93.56	90.31
22hd27963_1	3007402	2979448	451.11	420.12	93.13	97.82	93.52	90.61
22hd28061	3233550	3202530	485.03	447.55	92.27	97.78	93.45	90.18
22hd28179	2187268	2164404	328.09	314.38	95.82	97.61	93.04	89.93
22hd28028_1	2087224	2068252	313.08	292.49	93.42	97.6	92.97	89.91
22hd27859_1	2854704	2827716	428.21	403.15	94.15	97.41	92.51	90.36

22hd28114_1	2698598	2675490	404.79	375.01	92.64	97.73	93.34	88.98
22hd28115_1	2716524	2690892	407.48	381.84	93.71	97.56	92.87	89.47
22hd28072_1	2899886	2868316	434.98	396.49	91.15	97.9	93.75	89.16
22hd28085_1	2287508	2265216	343.13	324.22	94.49	97.55	92.89	89.18
22hd28076	2913554	2883232	437.03	406.28	92.96	97.92	93.69	90.44
22hd28034	3267046	3231268	490.06	456.99	93.25	97.63	93.1	90.05
22hd27884_3	2798408	2771214	419.76	392.31	93.46	97.73	93.29	90.83
22hd28066_1	3370278	3334532	505.54	475.66	94.09	97.77	93.42	89.33
22hd28040	2821910	2788816	423.29	389.51	92.02	97.77	93.41	88.40
22hd28168_1	3383298	3352124	507.49	474.65	93.53	97.63	93.09	89.73
22hd27874_1	3032406	2998676	454.86	417.60	91.81	97.81	93.55	90.57
22hd27831_1	2829230	2795762	424.38	398.62	93.93	97.45	92.69	90.31
22hd27908_2	2863136	2829666	429.47	407.82	94.96	97.63	93.1	90.59
22hd28002_1	3112016	3081796	466.80	433.06	92.77	97.69	93.22	89.52
B	2508984	2507590	376.35	348.75	92.67	99.27	97.69	93.12
D	2658196	2656860	398.73	371.53	93.18	99.2	97.46	93.24

Note: Effective Rate(%): Obtain the ratio of clean data to raw data after filtering; Q20、Q30: The percentage of total bases with Phred values greater than 20 and 30 after filtering; Coverage： The proportion of sequences obtained from sequencing to the entire genome.
