# Supplementary material for: QTL Mapping and Candidate Gene Screening for Enhancing Oil Content in Silage Maize
Source: Plants (Basel). 2025 Apr 10;14(8):1181. doi: 10.3390/plants14081181 (PMC12030292; doi:10.3390/plants14081181)
Supplement: Supplementary file 1 [file plants-14-01181-s001.zip › Supplementary Table 2.pdf]

Supplementary Table 2 Gene name and primer sequence

Gene ID	Sequence(5'~3')
ACT (J01238.1)	F:CAACAGAGAGAAAATGACGCAGA
	R:CACCTGAATCCATCACAATACCA
GRMZM2G133398	F:ATCATCCTCAGCCTCTCCATCTT
	R:GCCCATCAATATCTGCCTTTCCA
GRMZM2G156861	F:ATCCATCCTCCAGCCTCATCCATC
	R:AGCGACCTGACCTGACCTCAAG
GRMZM2G125268	F:AAACGACGGTATTGGATGTTATGAG
	R:CACCTCCCCTTGAATGATGTT
GRMZM2G002959	F:CATCCCTCCGTAGTTAGCTTCT
	R:CCTGTCGGCCAAGGCTATATAC
GRMZM2G343588	F:ACTCCAAGTCCAGATACACGAACC
	R:CCACTAGGCAATCCACTCATG
