# Supplementary material for: QTL Mapping and Candidate Gene Screening for Enhancing Oil Content in Silage Maize
Source: Plants (Basel). 2025 Apr 10;14(8):1181. doi: 10.3390/plants14081181 (PMC12030292; doi:10.3390/plants14081181)
Supplement: Supplementary file 1 [file plants-14-01181-s001.zip › Supplementary Table 3.pdf]

Supplementary Table 3 qRT-PCR amplification system

Components	Volume（μL）
cDNA	1
Primer F(10μM)	0.5
Primer R(10μM)	0.5
1×SYBR Green qPCR Mix	10
RNase -free water	8
Total	20
