# Supplementary figures and images for: QTL Mapping and Candidate Gene Screening for Enhancing Oil Content in Silage Maize
Source: Plants (Basel). 2025 Apr 10;14(8):1181. doi: 10.3390/plants14081181 (PMC12030292; doi:10.3390/plants14081181)

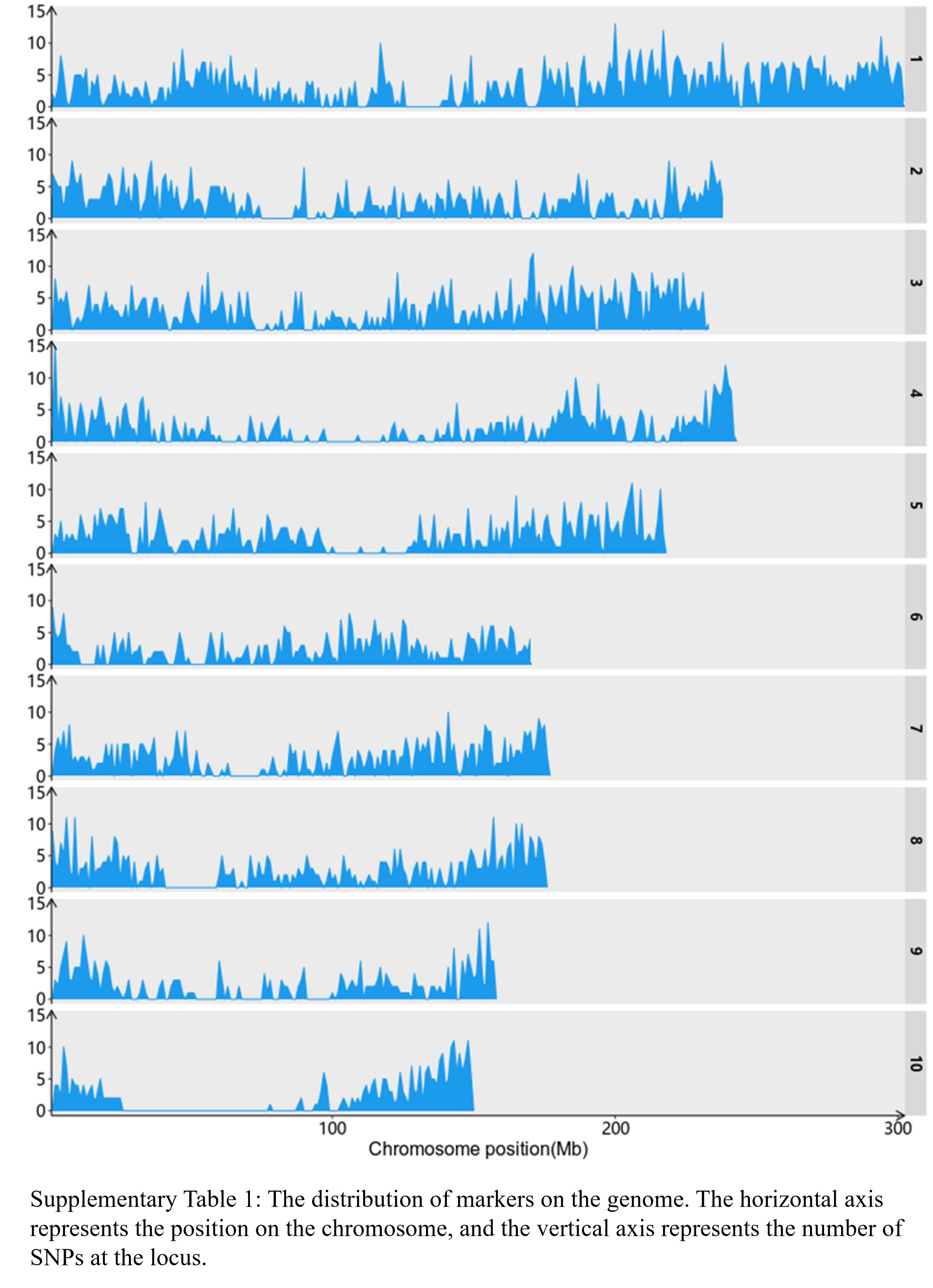

Supplement: Supplementary file 1 [file plants-14-01181-s001.zip › Supplementary Figure 1.jpg]

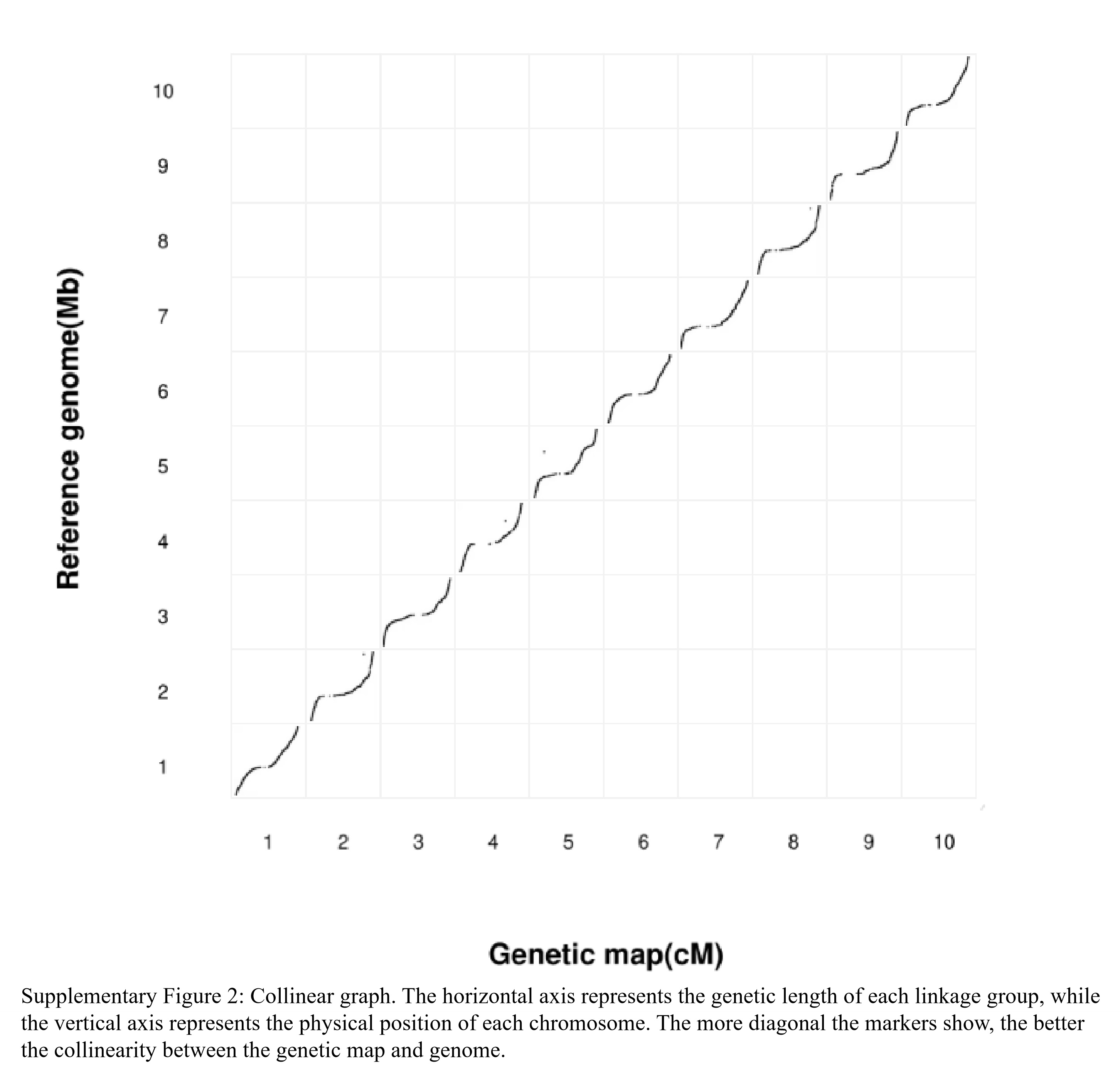

Supplement: Supplementary file 1 [file plants-14-01181-s001.zip › Supplementary Figure 2.jpg]

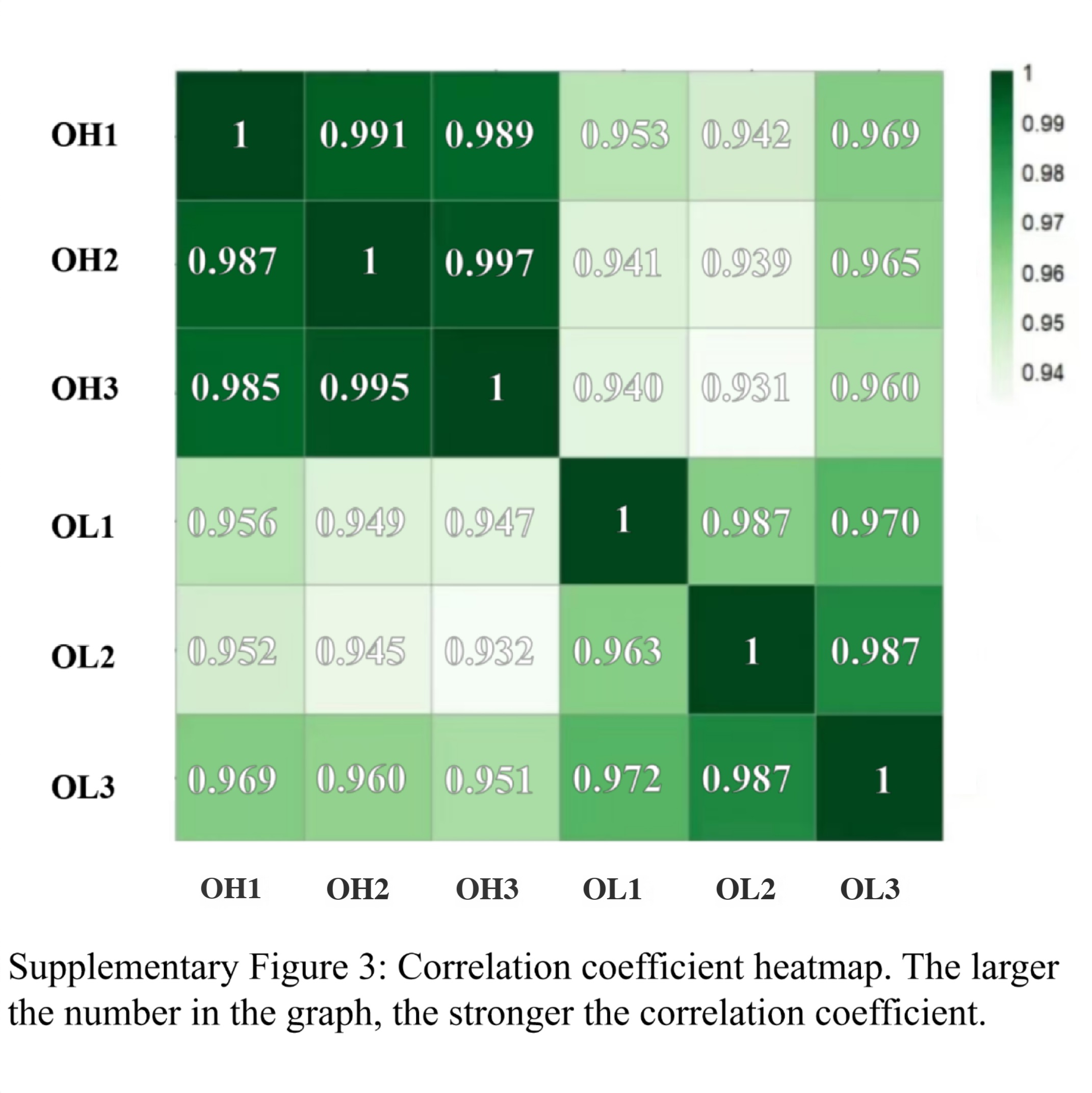

Supplement: Supplementary file 1 [file plants-14-01181-s001.zip › Supplementary Figure 3.jpg]
